# From Tumor Metastasis towards Cerebral Ischemia—Extracellular Vesicles as a General Concept of Intercellular Communication Processes

**DOI:** 10.3390/ijms20235995

**Published:** 2019-11-28

**Authors:** Xuan Zheng, Mathias Bähr, Thorsten R. Doeppner

**Affiliations:** Department of Neurology, University of Goettingen Medical School, 37075 Goettingen, Germany; xuan.zheng.1988@gmail.com (X.Z.); mbaehr@gwdg.de (M.B.)

**Keywords:** extracellular vesicles, stem cells, tumor formation, metastasis, neuroregeneration

## Abstract

Extracellular vesicles (EVs) have been tremendous carriers in both experimental and translational science. These vesicles—formerly regarded as artifacts of in vitro research—have a heterogeneous population of vesicles derived from virtually all eukaryotic cells. EVs consist of a bilayer lipid structure with a diameter of about 30 to 1000 nm and have a characteristic protein and non-coding RNA content that make up different forms of EVs such as exosomes, microvesicles, and others. Despite recent progress in the EV field, which is known to serve as potential biomarkers and therapeutic tools under various pathological conditions, fundamental questions are yet to be answered. This short review focuses on recently reported data regarding EVs under pathological conditions with a particular emphasis on the role of EVs under such different conditions like tumor formation and cerebral ischemia. The review strives to point out general concepts of EV intercellular communication processes that might be vital to both diagnostic and therapeutic strategies in the long run.

## 1. The History of Extracellular Vesicles (EVs)

In 1967, Wolf and co-workers discovered “platelet dust” in human plasma [1], and it was not long after, in 1969, when Anderson et al. identified this “platelet dust” as being involved in bone calcification processes of the epiphyseal cartilage [2]. In the 1970s, two independent research groups found virus-like particles in bovine serum, which were not identified at that time [3,4]. Later evidence from the 1980s showed that these vesicles were formed by the inpouring budding of intracellular endosomes that become multivesicular bodies (MVB) [5]. Nevertheless, initial interpretations still thought that EVs were cell debris only [6]. In 2007, however, a research group unraveled its intravesicular contents, indicating that EVs are not mere cell fragments, but contain a plethora of mRNAs and non-coding RNAs, which indicated a biological role of EVs under physiological conditions [7]. Further studies have shown that EVs are indeed involved in intercellular communication processes, exchanging proteins, lipids, and nucleic acids [8], which has increased the scientific interest in EVs and has even brought up the new International Society of Extracellular Vesicles (ISEV) [9]. The latter sets the standard of international EV research, publishing information, and guidelines for studies on EVs such as the positive marker of EVs [10,11,12,13] and the isolation methods [14,15,16,17]. Since EV enrichment still differs significantly between various research groups, the ISEV committee recommends using the general term “extracellular vesicles” (EVs) instead of “exosomes” or “microvesicles”. Hence, the present review will adhere to these recommendations, using the term EVs, although the majority of data available focus on exosomes.

## 2. Biogenesis of EVs

As stated previously, the ISEV defined the international standards of EV research and publishes the guidelines for the successful enrichment of EVs from various tissue sources. Exosomes are identified by their size, with diameters of around 30 to 150 nm, and biomarkers [18]. Since there is still no consensus about the specific marker of exosomes, some proteins that are deeply involved in exosome biogenesis are considered to be “specific” markers such as CD9, CD63, and CD81 [19]. The biogenesis of exosomes starts from forming early endosomes and becoming late endosomes or MVB [20]. As such, two major pathways for generating exosomes exist, i.e., the endosomal sorting complex required for the transport (ESCRT)-dependent pathway and the ESCRT-independent pathway [21]. The ESCRT-dependent pathway includes a complex of various proteins, which consist of four individual protein complexes, ESCRT-0 until ESCRT-III, as well as being associated with the AAA ATPase Vps4 complex [22]. It is believed that ESCRT-0 plays a role in cargo clustering in a ubiquitin-dependent manner.

On the contrary, ESCRT-I and ESCRT-II are responsible for the bud formation, whereas ESCRT-III drives vesicle scissions [23]. In the associated part, AAA ATPase Vps4 is the crucial protein that functions in endosomal protein trafficking, cytokinesis, and retroviral budding, which is also regulated and associated with ESCRT-III [24]. As mentioned previously, exosomes can also be formed in an ESCRT-independent pathway of which the mechanism is not yet precisely known. There is, however, evidence showing that CD63 is involved in the ESCRT-independent pathway, but the function of the latter is not clear either [25]. Along with this, ceramides might play a role in the ESCRT-independent pathway as well [26].

However, the biogenesis and biomarkers of microvesicles are quite different from exosomes. Microvesicles can be identified by their size (100 to 1000 nm) and specific biomarkers such as CD82, CD40, and Flotillin-2. While the microstructure between microvesicles and exosomes are quite similar, the functions of them are different. As such, only microvesicles, but not exosomes, can transfer plasmid DNA to recipient cells [27]. The formation of microvesicles begins with cellular activation and mobilization of cytosolic Ca^2+^ [28]. Several Ca^2+^ channels have been involved, such as transient receptor potential (TRP), calcium release-activated calcium channel protein-mediated store-operated channels and ryanodine receptors (RYR). When cells are at rest, the endoplasmic reticulum (ER) Ca^2+^ level is maintained at a higher level than the cytoplasm. During mobilization of Ca^2+^ into the cytoplasm, Ca^2+^ will be pumped out from ER into the cytoplasm through ER channels such as RYR and inositol trisphosphate receptors (IP3R).

Further research suggests that the interaction between TRPM7 and the calpain system could be a critical element in microvesicle biogenesis during Ca^2+^ mobilization. Following Ca^2+^ enrichment in the cytoplasm, aminophospholipid translocase (Flippase) is suppressed. While flippase is suppressed, scramblase is activated to promote a random distribution of phospholipids, which compromises membrane–cytoskeletal anchorage. In this context, combining intracellular hydrostatic pressure, contractile proteins, and BAR (Bin-Amphiphysin-Rvs) domain proteins results in the formation of membrane blebs. Finally, ESCRT and other proteins induce the scission of the vesicle neck and release microvesicles into the extracellular environment [29,30].

## 3. Tumor-Derived EVs in the Primary Tumor Site

Tumor cells exert various effects on surrounding cells in order to establish a tumor friendly microenvironment. Various evidence has demonstrated that tumor-derived EVs, such as exosomes contribute to tumor growth, immune inhibition, and metastasis [31]. Tumor-derived EVs modify the microenvironment of primary tumor sites, which contain different cell types, including endothelial cells, immune cells, and cancer-associated fibroblasts (CAFs) [32,33]. It is currently believed that tumor-derived EVs are not only a side phenomenon, but are likely to play a critical role in tumor formation and metastasis of different tumor cell types. Madeo and colleagues have found evidence that tumors initiate autonomic innervation called axonogenesis through the release of exosomes from these tumor cells [34]. Tumor-derived EVs will affect normal fibroblasts and convert them into CAFs like myofibroblasts, which are essential for cancer progression [35,36]. Similar research shows that melanoma cells directly affect the formation of the dermal tumor niche by releasing EVs containing miR-211, which directly target the insulin-like growth factor 2 receptor (IGFR2), leading to mitogen-activated protein kinases (MAPK) activation that encourages melanoma cell growth [37]. In peritoneal metastasis, tumor-derived EVs have been shown to turn formerly unaffected mesothelial cells into CAFs [38]. Expression of CAF specific protein markers in mesothelial cells, including fibroblast activation protein (FAP), alpha-smooth muscle actin (α-SMA), and fibronectin were increased and the expression of E-cadherin and vascular cell adhesion protein 1 was decreased after the treatment of mesothelial cells with associated malignant EVs [38]. Recent findings indicate a truncated and oncogenic form of the epidermal growth factor receptor (EGFR), known as EGFRVIII, which can be transferred between glioma cells by EVs. The latter then causes an increased anchorage-independent growth capacity and expression of anti-apoptotic genes [39]. Highly metastatic cancers, like ovarian cancer, secrete EVs carrying mRNA encoding for matrix metallopeptidase 1 (MMP1), which strongly induces metastatic behavior in moderately metastatic tumors [40]. Similar evidence shows S100 family members and caveolins promote metastasis in hepatocellular carcinoma cells where these proteins are highly enhanced in tumor-derived EVs [41].

Recent studies also indicated that CAFs, normal fibroblasts, and cancer cells could secrete exosomal miRNAs to affect each other [42]. For example, exosomes secreted by prostate cancer cells affect mesenchymal stem cells (MSCs) with respect to their adipogenic differentiation, distorting MSC differentiation towards alpha-smooth muscle actin (αSMA) positive myofibroblastic cells [43]. Furthermore, EVs derived from glioblastoma multiforme, which contain the vascular endothelial growth factor (VEGF) A, have been shown to disseminate both locally and at a distance. These EVs are significantly involved in increasing the permeability of endothelial–endothelial connections and are known to stimulate angiogenesis as well [44]. As such, lung cancer cells synthesize more miR-23a-rich exosomes under hypoxic conditions than do parental cells under normoxic conditions. The miR-23a, on the contrary, suppresses the tight junction protein ZO-1, which is one key element of EV-induced permeability of endothelial cells [45]. However, glioma cells enhance angiogenesis and inhibit endothelial cell apoptosis by releasing long non-coding RNA colon cancer-associated transcript 2 and POU class 3 homeobox 3 in their exosomes [46,47]. Further studies show that denervation in pancreatic ductal adenocarcinoma and gastric tumorigenesis can slow down the initiation and progression of cancer [48,49].

## 4. The Role of Tumor-Derived EVs in Tumor Metastasis

Angiogenesis and lymphangiogenesis are essential phenomena in metastasis. As mentioned before, the invasive nature of tumor cells located in the primary tumor site involves an affection of both blood vessels and lymphatic vessels. In this context, the exosomes “educate” from a distant, and at this stage, physiological cells still promote angiogenesis and lymphangiogenesis before metastasis. Melanoma-derived exosomes, for instance, induce vascular leakage at pre-metastatic sites and reprogram bone marrow progenitors towards a pro-vasculogenic phenotype from a distance [50]. In human renal cancer, cancer cells release CD105 positive exosomes, which stimulate angiogenesis and the formation of a lung premetastatic niche by significantly enhancing the expression of VEGFR1, VEGF, and MMP2 in CD146-sorted lung cells [51]. In hepatocellular carcinoma cells, hepatocellular carcinoma-derived exosomes contain miR-21, which will directly target phosphatase and the tensin homolog gene (PTEN), a well-known multi-functional tumor suppressor. The latter results in a reduction of phosphorylated levels of PTEN and in an increase of the expression of PTEN downstream inhibitory protein 3-phosphoinositide-dependent protein kinase-1 (PDK1) in hepatic stellate cells. The increased expression levels of PKD1 activate the PDK1/AKT signaling pathway in hepatic stellate cells. By activating the PDK1/AKT pathway, healthy hepatic stellate cells convert into CAFs [52]. Furthermore, colorectal cancer cells secret miR-25-3p exosomes that regulate the expression of VEGFR2, ZO-1, occludin, and Claudin5 in endothelial cells by targeting kruppel-like factors 2 and 4. These exosomes induce vascular leakage and enhance colorectal cancer metastasis in the liver and lung of mice [53].

Lymphangiogenesis induced by tumor cells contributes to the metastatic spread towards the lymph nodes and beyond to distal organs [54]. Some cancer types, such as rhabdomyosarcoma, have a high propensity for lymph node metastasis [55]. In hepatocarcinoma cells, exosomes containing elevated C-X-C chemokine receptor type 4 from highly lymph node metastatic mouse hepatocarcinoma Hca-F cells were able to promote the migration and invasion of paired syngeneic Hca-P cells that have a low metastatic potential [56]. In addition, cervical squamous cells secrete exosomes, which contain miR-221-3p that promote lymphangiogenesis and lymphatic metastasis by targeting vasohibin 1. The latter functions as a negative regulator of lymphangiogenesis [57].

## 5. The Role of Tumor-Derived EVs in Epithelial-Mesenchymal Transition (EMT)

EMT is one of the critical processes of metastasis, which is characterized by the loss of epithelioid features and the acquisition of mesenchymal-like features, including cell migration and invasion [58]. Evidence shows that tumor-derived EVs carrying hypoxia-inducible factor 1-alpha (HIF1α) and latent membrane protein 1 (LMP1) are significantly more often associated with increased cancer migration and enhanced invasiveness [59,60]. Glioma-associated human MSCs can transfer miR-1587 to tumor-initiating glioma stem-like cells (GSC) by releasing exosomes that increase proliferation and clonogenicity, which in turn enhances the aggressiveness of glioblastoma cells [61]. Furthermore, EVs from early-stage breast cancer cells containing high levels of miR-105 primarily target ZO-1 to destroy vascular endothelial barriers to promote metastasis even further [62].

## 6. Tumor-Derived EVs Regulate Immune Responses

Immune escape is an essential factor for tumor formation, both in primary tumor sites and in pre-metastatic niches. Tumors escape immune surveillance by modulating the recruitment and expansion of immunosuppressive cell populations in the primary tumor site or the pre-metastatic niche, and switch the phenotype as well as the function of healthy immune cells into a tumor-promoting state [63].

Tumor-derived EVs from tumor patients can down-regulate health of CD3ζ donors, which is part of the T-cell antigen receptor and JAK3 (Janus kinase 3) expression in T cells. The latter mediates Fas/FasL (Fas ligand)-driven apoptosis of CD8+ T-cells. Furthermore, these exosomes decrease the cytotoxic activity of NK cells as well [64]. In addition, exosomes derived from murine mammary adenocarcinomas can induce healthy bone marrow myeloid cells to differentiate into myeloid-derived suppressor cells (MDSCs), which can promote tumor progression by suppressing T cell activation [65]. Furthermore, tumor-derived EVs can inhibit the signaling and proliferation of activated CD8+ cells, as well as induce apoptosis of CD8+ cells, thus suppressing cytotoxic T cells with subsequent immune escape [66]. Because they can secrete miR-214 enriched EVs, tumors can thus increase the population of CD4+CD25highFoxp3+ regulatory T cells (Tregs) in order to suppress the immune response [67]. Pancreatic cancer cells will secrete miR-212-3p enriched exosomes. These pancreatic cancer-derived exosomes inhibit the expression of regulatory factor X associated protein (RFXAP), an important transcription factor for MHC II, in dendritic cells (DCs), to induce immune tolerance [68]. Another research paper shows that pancreatic cancer derived exosomes can down-regulate toll like receptor 4 on DCs and downstream cytokines in DCs via miR-203 [69].

Tumor cells also evade immune surveillance by up-regulating the surface expression of programmed death-ligand 1 (PD-L1), which interacts with a programmed death-1 (PD-1) receptor on T cells to elicit an immune checkpoint response. A recent study indicates that these cancer cells secret PD-L1 positive exosomes into the blood like drone-targeting T cells, which fatigue T cells before they reach the cancer cells [70]. Mingqi and his co-workers have recently published an article that reported that not only PD-L1, but also anti-tumor immunity is present in exosomes secreted by tumor cells. By knocking out Rab27a or using the inhibitor GW4869, this treatment can inhibit the secretion of exosomes containing PD-L1 and thus significantly affect tumor disease [71].

## 7. Ischemic Stroke and Neuroregeneration as an Intriguing Therapeutic Target

Stroke is a well-known disease in developed and developing countries. It has become the third major cause of long-term disability and death in the past ten years [72]. Despite recent progress in clinical stroke treatment with intravenous thrombolysis and the advent of endovascular thrombectomy [73], the majority of patients do not qualify for either treatment paradigm. Consequently, adjuvant treatment paradigms have been studied extensively in the last decades. Since neuroprotective drugs have failed in clinical trials until recently [74,75,76], the scientific focus has now switched from neuroprotection towards neuroregeneration. The basis of the latter is the persisting neurogenesis that is also found in the adult mammalian brain [77,78]. Further evidence has shown that neurogenesis persists in distinct regions of the brain, among which is the subventricular zone (SVZ) of the lateral ventricles [79,80,81,82]. The SVZ hosts stem cell-like cells that are stimulated upon induction of cerebral ischemia, proliferating and migrating towards the site of lesion where some of these cells develop into mature neurons [83,84,85]. Unfortunately, both the survival rate and the differentiation rate of these endogenous stem cells are limited by the harsh microenvironment in the stroke area [86,87]. While the underlying mechanisms that lead to this condition are complex and are not yet fully understood, endogenous neurogenesis can be boosted via the transplantation of ectopic adult stem cells like neural progenitor cells (NPCs) or MSCs from different tissue sources [88,89]. Bone marrow MSC (BM-MSC) transplantation can reduce neurological deficits after a stroke in short-term or long-term stroke models [90,91]. In line with this, MSCs from other cell sources such as embryonic stem cells, blood, placenta, or adipose tissue can improve functional outcomes in the stroke model as well [92,93,94,95]. The grafted MSCs can induce angioneurogenesis and axonal plasticity as well as a modulation of immune responses [96]. Recent research demonstrates intravenous delivery of exogenous NPC transplantation can stabilize the blood–brain barrier, improve neurological outcomes, induce angioneurogenesis, and axonal plasticity. Moreover, these grafted NPCs can modulate immune responses similar to MSCs [97,98]. Indeed, some randomized clinical trials, such as the MASTERS (Safety and efficacy of multipotent adult progenitor cells in acute ischaemic stroke) trial, using intravenous multipotent adult progenitor cells have already been performed, although a clear and distinct impact on neurological recovery is still missing [99].

## 8. Stem Cells Act in a Paracrine Way

Even though ectopic stem cells induce a better outcome after stroke, as shown before, these cells virtually face the same fate as endogenous stem cells, i.e., depicting low survival rates and low differentiation rates [100]. Moreover, research about the distribution of systemic administration mesenchymal stem cells showed that most of the MSCs are stuck in the lung, liver, spleen, or kidney. The number of MSCs that reached the brain is limited [101]. In our previous study, different administration methods could affect the outcome of neural progenitor cell transplantation [102]. Interestingly, it appears evident that, rather than the stem cells themselves, their secretion products are responsible for these effects. Indeed, evidence suggests that the condition medium derived from SVZ-derived NPCs induce long-term post-ischemic neuroregeneration and improves neurological recovery after stroke [103]. This process has been found to involve a diversity of signaling cascades, among which are Bcl-2, pAkt, and pSTAT-3 [103]. Whereas earlier research was in favor of soluble factors, derived from stem cells, as being the critical mediators of stem cell-induced effects, more recent data is in favor of EVs being the key players [102,104,105].

## 9. EVs as Therapeutic Tools for Stroke Treatment

Recent studies show that EVs from stem cells may be a critical factor in achieving neuroregeneration and angiogenesis. By the systemic administration of MSC-derived exosomes, neurogenesis and angiogenesis are significantly increased along the ischemic boundary zone of the cortex and striatum in a rat stroke model [106]. Furthermore, MSC-derived exosomes improve cognitive impairment, synaptic transmission, and long-term potentiation after transient global cerebral ischemia [107]. In this context, previous research of our own systematically compared the effects of human bone marrow-derived MSC-EVs (BM-MSC-EVs) with MSCs themselves. These data showed that MSC-EVs induced long-term neuroprotection, promoted neuroregeneration, and increased neurological recovery, all of which are most likely the consequence of modulation of the peripheral post-stroke immune responses [108]. Of note, it is worth mentioning that different MSC sources also show the same effect, such as human adipose-derived MSC-EVs [109,110] and human umbilical cord blood stem cell-derived exosomes [111] in the stroke model.

Stem cell-derived EVs modulate neurogenesis and angiogenesis by the kind of cargo they carry, such as mRNAs and proteins. For example, a recent study indicated that BM-MSC-EVs can upregulate Akt phosphorylation and Bcl-2 expression, downregulate Bax expression, and reduce the cleavage of caspase-3 in order to protect pheochromocytoma PC12 from glutamate-induced injury [112]. BM-MSCs transfer miR-133b to neurons to increase neurite branch numbers and total neurite length via exosomes [113]. Grafted stem cells can enrich secreted EVs with IFN-γ in order to modulate immune responses in the recipient through IFN-γ/Ifngr1 complexes [114]. In line with this, research done by Zhang et al. used miR-17-92 overexpressing MSC-EVs to treat cortical neurons, indicating that, compared to native MSC-EVs, the overexpressing miR-17-92 exosomes can promote axonal growth in a more obvious way [115].

## 10. Glial Cell-Derived EVs under Stroke Condition

The majority of neural cells located in the brain are not the consequence of neurons, but are due to the sheer number of glial cells, including oligodendrocytes, astrocytes, ependymal cells, and microglia. These cells, and their EVs, play an essential role in supporting neuron functions and intercellular communication under physiology conditions [116]. Under pathological conditions, however, the role of these EVs have to be regarded differentially. For example, microglia will act as macrophages inside the brain at the acute phase of the stroke. Extracellular ATP from damaged cells will activate P2X7 receptors on microglia. Activated microglia will, in turn, release EVs which contain mature cytokine interleukin-1β (IL-1β), yielding acute inflammatory responses [117]. When microglia are stimulated by the “eat me” signal [118,119,120], the size and cargos of microglia-derived EVs will be changed, in comparison to the normal condition. There is evidence showing, that when treated with LPS, microglia released larger EVs (200 to 300 nm) compared to the control group. These EVs were enriched with TNF and IL-6, and the inhibition of the TNF pathway significantly reduced this process [121]. Another study showed that microglia secreted nicotinamide phosphoribosyltransferase (NAMPT), which is a protein that stimulates pro-inflammatory signaling cascades via EVs upon the induction of pathological conditions like ischemia [122].

Apart from microglia, EVs also play pivotal roles in astrocytic cell function under stroke conditions. Indeed, astrocytes can act like nonprofessional phagocytic cells [123,124]. Upon this process, reactive astrocytes upregulate ABCA1, MEGF10, and GULP1, which are required for phagocytosis under stroke conditions [125]. However, astrocytes have been shown to also contain EVs that carry the cellular prion protein (PrPc) under normoxic conditions. PrPc, in turn, can protect neural cells against oxidative stress, hypoxia, ischemia, and hypoglycemia in order to improve the survival rate of these cells [126,127,128]. Even though the underlying mechanisms are not yet understood, some research suggests that prostaglandin D2 synthase might be involved in PrPc-mediated neuroprotection [129]. More recent in vitro research using a model of oxygen-glucose-deprivation (OGD) has shown that astrocytes promote cell survival by transferring functional mitochondria to neurons and vice versa via the secretion of EVs [130]. 

Whereas a decent amount of data regarding EVs derived from astrocytes and even more so from existing microglia, information on oligodendrocyte-derived EVs under conditions of hypoxia or cerebral ischemia is scarce. Some reports, however, suggest that oligodendrocytes promote neuronal survival during OGD by transferring superoxide dismutase and catalase into neurons via exosomes [131].

## 11. Stem Cell-Derived EVs and Tumor-Derived EVs

Stem cells and tumors or cancer cells are completely different cells, but they share the same EV biogenesis pathway. As previously described, grafted stem cells under conditions of pre-clinical stroke act in a paracrine way by secreting EVs. The purpose of tumor-derived EVs, however, is to create a pre-metastasis niche distant. As such, EVs from stem cells might have similar purposes—to create a “pre-metastasis niche” for endogenous neural stem cells from the SVZ. As previously mentioned, astrocytes can act as nonprofessional phagocytic cells [123,124], while MSC-derived EVs can ameliorate inflammation-induced astrocyte alterations in mice [132]. Interestingly, astrocytes previously exposed to cerebral ischemia of rats were transformed to release protective EVs rather than harmful pro-inflammatory EVs, provided that these astrocytes were first treated with MSC-EVs [133]. The research on the impact of MSC-EVs’ on microglia under stroke conditions is still limited, but other research fields such as spinal cord injury give us some ideas. MSC-EVs can modify the microglial response and even improve clinical outcomes [134]. Along with this, EVs from umbilical cord MSCs can reduce microglia-mediated neuroinflammation in perinatal brain injury [135]. 

Stem cell-derived EVs can also shift pro-inflammatory M1 polarized microglia towards M2 microglia, which can attenuate the neuroinflammatory response in rats exposed to traumatic brain injury [136]. What makes the correlation between stem cell-derived EVs and tumor-derived EVs even stronger is the fact that MSC-derived EVs have been demonstrated to promote cell growth and mobility of non-small cell lung cancer cells [137]. Furthermore, these same EVs also enhance the proliferation and migration of breast cancer cells [138]. Collectively, the above results suggest that similar mechanisms that are responsible for stem cell-derived EVs remodeling of the post-ischemic microenvironment, in order to enhance endogenous neuroregeneration, are also, at least in part, responsible for tumor formation and metastasis due to tumor-derived EVs. By further understanding these mechanisms, we can thus better understand the mechanisms underlying neuroprotection induced by stem cell-derived EVs and vice versa.

## 12. Conclusions and Outlook

EVs have made a tremendous career from being regarded as only cell debris, towards a general concept of intercellular communication processes under both physiological and pathological conditions. The precise role of EVs under such different conditions like tumor formation and cerebral ischemia is just beginning to be understood. New insights of underlying mechanisms on how EVs can precisely induce the immune escape of tumor cells, lymphangiogenesis, and angiogenesis, might provide therapeutic targets or tools in the future. Along with this, stem cell-derived EVs might be a potential therapeutic tool of choice for the treatment of stroke or other diseases. However, more evidence with regard to the underlying mechanisms and safety issues of EVs from either cell source, whether it is a stem cell or tumor cell, is urgently needed. During such an endeavor, deciphering common pathways and characteristics of these aforementioned EVs will greatly support the contemporary EV field.
